# The Protective Effect of a Unique Mix of Polyphenols and Micronutrients against Neurodegeneration Induced by an In Vitro Model of Parkinson’s Disease

**DOI:** 10.3390/ijms23063110

**Published:** 2022-03-13

**Authors:** Francesca Pacifici, Chiara Salimei, Donatella Pastore, Gina Malatesta, Camillo Ricordi, Giulia Donadel, Alfonso Bellia, Valentina Rovella, Marco Tafani, Enrico Garaci, Manfredi Tesauro, Davide Lauro, Nicola Di Daniele, David Della-Morte

**Affiliations:** 1 Department of Systems Medicine, University of Rome “Tor Vergata”, 00133 Rome, Italy; francesca.pacifici@uniroma2.it (F.P.); chiara.salimei@gmail.com (C.S.); bellia@med.uniroma2.it (A.B.); valerovix@yahoo.it (V.R.); mtesauro@tiscali.it (M.T.); d.lauro@med.uniroma2.it (D.L.); didaniele@med.uniroma2.it (N.D.D.); 2 Department of Human Sciences and Quality of Life Promotion, San Raffaele University, 00166 Rome, Italy; d.pastore3@inwind.it (D.P.); enrico.garaci@sanraffaele.it (E.G.); 3 Department of Biomedicine and Prevention, University of Rome Tor Vergata, 00133 Rome, Italy; G.malatesta@outlook.it; 4 Cell Transplant Center, Diabetes Research Institute, School of Medicine, University of Miami Miller, Miami, FL 33136, USA; ricordi@miami.edu; 5 Department of Clinical Sciences and Translational Medicine, University of Rome Tor Vergata, 00133 Rome, Italy; donadel@uniroma2.it; 6 Department of Medical Sciences, Fondazione Policlinico Tor Vergata, 00133 Rome, Italy; 7 Department of Experimental Medicine, Sapienza University of Rome, 00161 Rome, Italy; marco.tafani@uniroma1.it; 8 Department of Neurology, Evelyn F. McKnight Brain Institute, Miller School of Medicine, University of Miami, Miami, FL 33136, USA; 9 Interdisciplinary Center for Advanced Studies on Lab-on-Chip and Organ-on-Chip Applications (ICLOC), University of Rome Tor Vergata, 00133 Rome, Italy

**Keywords:** SIRT1, polyphenols, Parkinson’s disease, inflammation, oxidative stress

## Abstract

Parkinson’s disease (PD) is second-most common disabling neurological disorder worldwide, and unfortunately, there is not yet a definitive way to prevent it. Polyphenols have been widely shown protective efficacy against various PD symptoms. However, data on their effect on physio-pathological mechanisms underlying this disease are still lacking. In the present work, we evaluated the activity of a mixture of polyphenols and micronutrients, named A5^+^, in the murine neuroblastoma cell line N1E115 treated with 6-Hydroxydopamine (6-OHDA), an established neurotoxic stimulus used to induce an in vitro PD model. We demonstrate that a pretreatment of these cells with A5^+^ causes significant reduction of inflammation, resulting in a decrease in pro-inflammatory cytokines (IFN-γ, IL-6, TNF-α, and CXCL1), a reduction in ROS production and activation of extracellular signal-regulated kinases (ERK)1/2, and a decrease in apoptotic mechanisms with the related increase in cell viability. Intriguingly, A5^+^ treatment promoted cellular differentiation into dopaminergic neurons, as evident by the enhancement in the expression of tyrosine hydroxylase, a well-established dopaminergic neuronal marker. Overall, these results demonstrate the synergic and innovative efficacy of A5^+^ mixture against PD cellular pathological processes, although further studies are needed to clarify the mechanisms underlying its beneficial effect.

## 1. Introduction

The significant increase in life expectancy has led to a rise in all chronic diseases, including neurodegenerative illnesses. Among those, Parkinson’s disease (PD) is the second-most common neurodegenerative disorder [1]. The main characteristic of PD is the loss of dopaminergic neurons in the *substantia nigra pars compacta* [2], which induces both motor (tremor, postural instability and slower movement) and non-motor dysfunctions (anxiety and depression among others) in affected patients [3,4]. The physiopathology of PD is complex and multifactorial [5]. A condition of chronic neuroinflammation is one of the main contributing mechanisms related to the onset and progression of the disease [5]. Increased levels of several pro-inflammatory cytokines and chemokines such as tumor necrosis factor (TNF)-α; Interferon (IFN)-γ; and Interleukin (IL)-1β, IL-2, and IL-6 have been reported [6,7]. This pro-inflammatory condition has been linked with a direct increase in reactive oxygen species (ROS) production [8], and in turn, with apoptotic damage in neurons, as demonstrated by an increase of pro-apoptotic factors, such as caspase-3, both in post-mortem PD patients and PD animal models [9].

Therapeutic strategies for PD care nowadays show several side effects and sometimes low efficacy in counteracting PD symptoms. Thus, preventive strategies to slow down the progression of disorder and/or reduce both motor and non-motor symptoms, also avoiding side effects, are necessary, even to improve the quality of life in PD patients.

Natural antioxidant and anti-inflammatory compounds, such as resveratrol, have been reported to have neuroprotective effects against neurodegenerative disorders [10,11]. Among those, resveratrol administration improves the motor and cognitive function and reduces oxidative and apoptotic damage in animal models of PD [12,13,14]. However, the limitation for many of these compounds, such as resveratrol, is the low bioavailability and high susceptibility to oxidation [10]. Polydatin is a glycosylated derivative with high bioavailability compared to resveratrol and other polyphenols [10]. Interestingly, polydatin already showed neuroprotective efficacy without side effects either in in vivo or in vitro studies [15,16]. In particular, in an in vitro model of PD, it has been reported that polydatin promotes cell viability and reduces apoptosis and ROS production [16]. Similarly to polydatin, pterostilbene is a compound derived from methoxylation of resveratrol with high bioavailability and, along with honokiol and ellagic acid, has already reported strong neuroprotective effects [10,17,18,19,20,21]. However, data on these polyphenols on protective mechanisms against PD are still sparse.

Based on all these findings, in the present study, we tested the potential neuroprotective effect of a natural patented compound, known as A5^+^ [22], a combination of both polydatin and pterostilbene and other beneficial micronutrients mixed together, in an in vitro model of PD. Recently, A5^+^ mixture has been demonstrated to strongly protect against influenza A virus, and SARS-CoV-2 by a reduction in virus-mediated inflammation [22].

We pretreated murine neuroblastoma cell line N1E115 with A5^+^ and then induced a PD model by the addition of an established neurotoxic stimulus, 6 Hydroxydopamine (6-OHDA) [23]. We demonstrated that pre-treatment with A5^+^ significantly blunted the inflammatory response and apoptosis induced by 6-OHDA. We also reported the antioxidant properties of A5^+^, associated with a reduction in ROS production and activation of extracellular signal-regulated kinases (ERK)1/2. A significant dopaminergic neuronal differentiation after treatment with A5^+^ was also present. All these results suggest that A5^+^, by acting at different levels involved in pathogenesis of PD, may be useful in counteracting the onset and progression of PD.

## 2. Results

### 2.1. Evaluation of Neurotoxicity Induced by 6-OHDA and A5^+^ in N1E115

**Figure 1:**
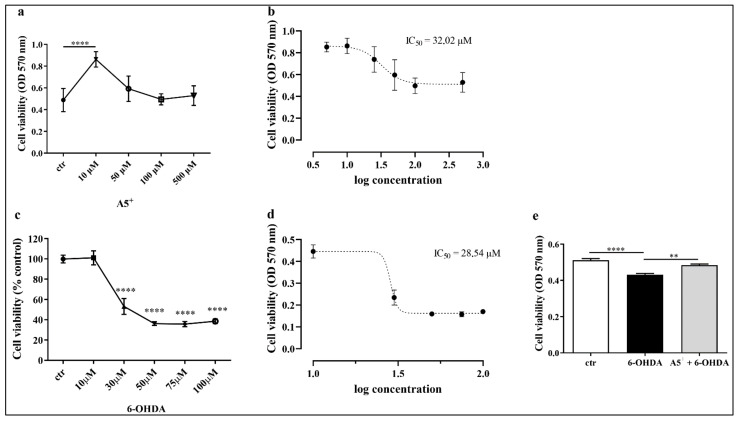
Cell viability assessment following A5^+^ and/or 6-OHDA administration. (**a**) Cell viability assessment following different concentrations of A5^+^ administration; (**b**) IC_50_ related to A5^+^. (**c**) Cell viability assessment following different concentrations of 6-OHDA administration; (**d**) IC_50_ related to 6-OHDA. (**e**) Cell viability following 6-OHDA (black bar) or A5^+^ + 6-OHDA (grey bar) administration. Data are reported as mean ± SEM (*n* = 4). (** *p* < 0.005; **** *p* < 0.0001). ctr: control; 6-OHDA: 6-hydroxydopamine; OD: optical density.

In order to establish the right concentration of A5^+^ to use for the further experiments, we treated murine neuroblastoma cell line N1E115 with different doses (10, 50, 100 and 500 μM) of A5^+^ for 48 h (h). Then, cell viability was assessed by using MTT assay as reported in Materials and Methods section. As shown in Figure 1a, treatment with A5^+^ did not impact negatively on cell viability, except a significant increase at 10 μM compared to control cells (*p* < 0.0001). Moreover, we also established the IC_50_ for this compound; as shown in Figure 1b, the IC_50_ for A5^+^ was about 3202 μM. Then, to avoid side effects, we used a concentration of A5^+^ lower that 30 μM, and based on cell viability assay, we used 10 μM of A5^+^ for all further experiments. These results also align with the findings of De Angelis et al. [22].

Similarly, we also tested 6-OHDA toxicity; N1E115 cells were treated with different concentrations of 6-OHDA (10, 30, 50, 75 and 100 μM) for 24 h, and then cell viability was evaluated by performing MTT. As shown in Figure 1c cell viability significantly decreased in a dose-dependent manner following 6-OHDA administration; in particular, cell viability at 30 μM of 6-OHDA was nearly 50%, as reported by the IC_50_ analysis (Figure 1d). Thus, this concentration will be employed for the following experimental procedures.

Once the concentrations for A5^+^ and 6-OHDA were determined, we evaluated whether A5^+^ protects against 6-OHDA’s neurotoxic effect. We pretreated cells with selected concentrations of A5^+^ for 48 h. Then, 6-OHDA was added together with freshly prepared A5^+^ for 24 h. As reported in Figure 1e, 6-OHDA reduced the cell viability compared to control cells (*p* < 0.0001), which was partially, but significantly, restored by A5^+^ pre-treatment (*p* < 0.005). These data suggest that the right dose of A5^+^ exerts a protective effect against neurotoxicity induced by 6-OHDA.

### 2.2. A5^+^ Protects from 6-OHDA-Iduced Neuroinflammation

**Figure 2:**
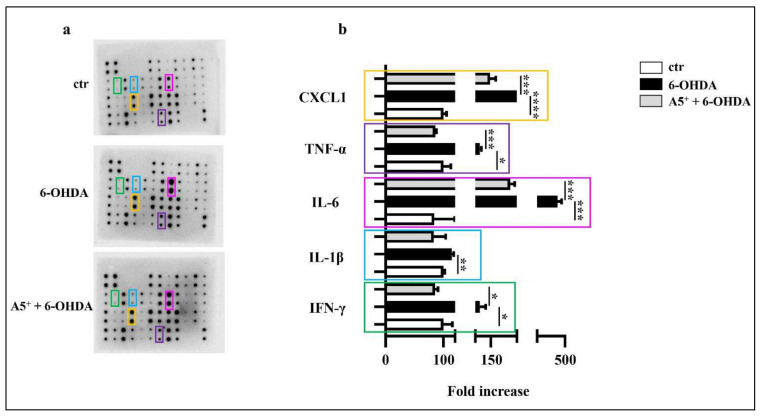
A5^+^ reduced neuroinflammation induced by 6-OHDA. (**a**) Representative blot assay reporting all cytokines evaluated; (**b**) Bar graph illustrating the most relevant cytokines modulated by both 6-OHDA and A5^+^ administration. Data are reported as mean ± SEM (*n* = 4). (* *p* < 0.05; ** *p* < 0.005; *** *p* < 0.001; **** *p* < 0.0001). ctr: control; 6-OHDA: 6-hydroxydopamine; IFN-γ: Interferon-γ; IL-1β: Interlukin-1β; TNF-α: Tumor Necrosis Factor-α; CXCL1: chemokine (C-X-C motif) ligand 1.

Inflammation, characterized by an increased cytokines release, is a typical future neurodegeneration process, including PD [24]. 6-OHDA already promotes an inflammatory response in an in vitro model of PD [25]. According to these findings, we evaluated the effect of 6-OHDA on inflammation in our cellular model. Several cytokines were analyzed, with particular attention to those that are modulated in PD. As reported in Figure 2, 6-OHDA administration significantly increased the release of IFN-γ (*p* < 0.05), IL-1β (*p* < 0.01), IL-6 (*p* < 0.001), and TNF-α (*p* < 0.05) compared to control cells. Moreover, chemokine CXCL1 levels also increased in 6-OHDA-treated cells (*p* < 0.0001). Then, we demonstrated that pretreatment with A5^+^ significantly reduced the levels of IFN-γ (*p* < 0.05), IL-1β (not significant), IL-6 (*p* < 0.001), TNF-α (*p* < 0.001), and CXCL1 (*p* < 0.001) compared to cells treated only with 6-OHDA. These data are in agreement with our previous data demonstrating that A5^+^ reduced IL-6 production in Vero E6 cells infected with Influenza A Virus [22], and further suggest relevant anti-inflammatory properties of A5^+^.

### 2.3. A5^+^ Protects from 6-OHDA-Iduced Apoptotic Death

**Figure 3:**
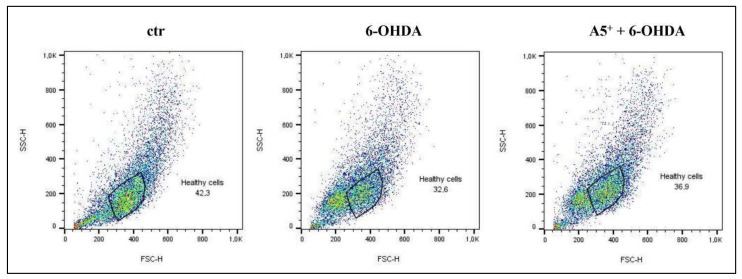
Cell shrinkage induced by 6-OHDA. Cytofluorimetric analysis reporting the alteration in cell structure and dimension following the neurotoxic insult. ctr: control; 6-OHDA: 6-hydroxydopamine.

6-OHDA stimulation reduces cell survival and similar findings have been reported in human neuroblastoma cell line SH-SY5Y [26]. Since during apoptosis a cell shrinkage occurs, characterized by a reduction in both forward scatter (FSC), which represent cell diameter, and side scatter (SSC), that reflects intracellular structures [27], we firstly analyzed cells morphology by FACS analysis. According to our hypothesis, treatment with 6-OHDA reduced both FSC and SSC compared to no treated cells, suggesting the induction of apoptosis (Figure 3). Pretreatment with A5^+^ partially recovers the cellular structure and dimension compared to cells treated only with 6-OHDA (Figure 3).

**Figure 4:**
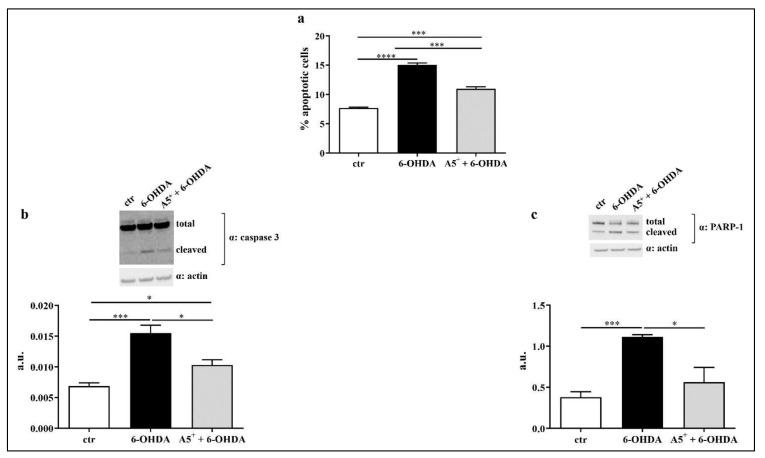
A5^+^ blunted 6-OHDA-induced apoptosis. (**a**) Sub-G1 phase assessment by cytofluorimetric analysis. (**b**) Caspase 3 cleavage. (**c**) PARP-1 activation. Data are reported as mean ± SEM. Graphs represent one of three separate studies, all yielding similar results (*n* = 4). (* *p* < 0.05; *** *p* < 0.001; **** *p* < 0.0001). ctr: control; 6-OHDA: 6-hydroxydopamine. a.u. = arbitrary unit.

To further confirm the presence of apoptosis in our model, we analyzed the Sub-G1 phase by treating cells with Propidium Iodide as described in the Materials and Methods section. As reported in Figure 4a, 6-OHDA significantly induced apoptotic death compared to control non-treated cells (*p* < 0.0001). According to cell recovery observed in Figure 3, A5^+^ pretreatment significantly blunted 6-OHDA-induced apoptosis (*p* < 0.001). We then analyzed the activation of both caspase 3 and PARP-1, which are well-established mediators of apoptotic death [28]. As expected, caspase 3 cleavage significantly increased in 6-OHDA-treated cells compared to ctr cells (*p* < 0.001), and A5^+^ pretreatment further blunted this cleavage (*p* < 0.05). Similarly, PARP-1 activation also increased following 6-OHDA administration compared to ctr cells (*p* < 0.001), and A5^+^ further reduced PARP-1 cleavage (*p* < 0.05). These findings highlight the anti-apoptotic effect of A5^+^ against neurotoxic insult.

**Figure 5:**
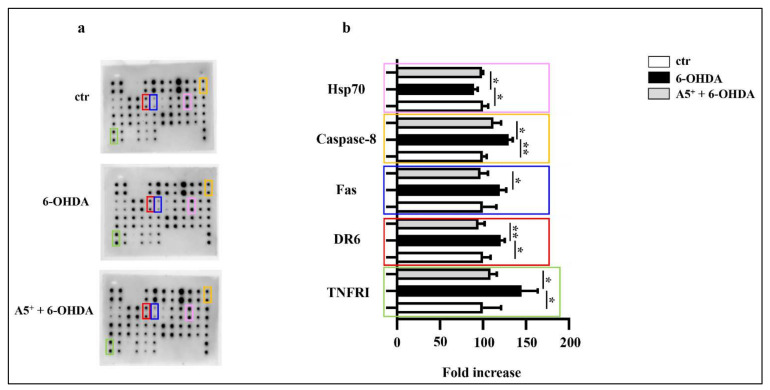
A5^+^ blunted the death-receptor-induced apoptotis. (**a**) Representative blot assay reporting all factors evaluated; (**b**) Bar graph illustrating the most relevant factors modulated by both 6-OHDA and A5^+^ administration. Data are reported as mean ± SEM (*n* = 4). (* *p* < 0.05; ** *p* < 0.005). ctr: control; 6-OHDA: 6-hydroxydopamine; TNFRI: Tumor Necrosis Factor Receptor I; DR6: Death Receptor 6; Hsp70: Heat Shock Protein 70.

Dopaminergic neurons, during the development of PD, increase the expression of several death receptors, mainly belonging to the TNF superfamily, which contribute to pro-inflammatory and apoptotic damage [29,30]. Among those, in particular, an increase in the levels of Fas, TNFRI, and DR6 were found, which promote caspase 8 activation and ultimately lead to apoptosis [29,30]. Then, by using our model, we analyzed the expression levels of several molecules involved in PD-related apoptosis, especially focusing on the death receptor pathway. As reported in Figure 5, 6-OHDA administration significantly increased TNFRI (*p* < 0.05), DR6 (*p* < 0.05), and caspase-8 (*p* < 0.005) levels compared to control cells, while Fas enhancement was not significant, although a slight increase was observed after 6-OHDA treatment. Moreover, Hsp70 expression decreased following 6-OHDA treatment (*p* < 0.05). According to previous results, A5^+^ administration significantly reduced TNFRI (*p* < 0.05), DR6 (*p* < 0.005), caspase-8 (*p* < 0.05), and Fas levels (*p* < 0.05), and partially restored Hsp70 (*p* < 0.05) compared cells treated only with 6-OHDA, further confirming the anti-apoptotic and neuroprotective role of A5^+^.

### 2.4. A5^+^ Reduces ROS Production and Increases ERK1/2 Activation

**Figure 6:**
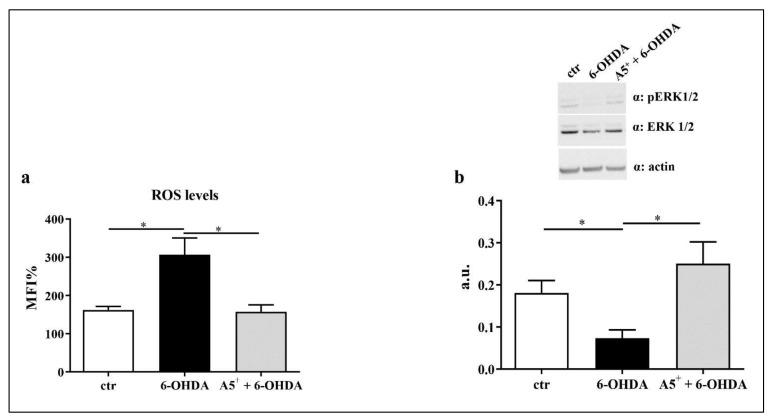
A5^+^-mediated antioxidant effect. (**a**) ROS production assessed by cytofluorimetric analysis. (**b**) pERK/1/2 levels. Data are reported as mean ± SEM. Graphs represent one of three separate studies, all yielding similar results (*n* = 4). (* *p* < 0.05). ctr: control; 6-OHDA: 6-hydroxydopamine. a.u. = arbitrary unit.

Neuroinflammation and ROS production are deeply linked in contributing to neuronal damage [8]. 6-OHDA administration significantly enhances intracellular levels of ROS in the human neuroblastoma cell line [31]. Therefore, we measured ROS levels in our in vitro model. As reported in Figure 6a, cells treated with 6-OHDA showed increased intracellular ROS levels compared to control cells (*p* < 0.05). As expected, A5 ^+^ significantly reduced ROS production compared to 6-OHDA-treated cells (*p* < 0.05). According to previous findings found in a similar study [32], we also observed an increase in pERK1/2 activation, a pivotal pathway in the regulation of ROS production [33], following A5^+^ administration, compared to cells treated with only 6-OHDA (*p* < 0.05) (Figure 6b). These data suggest that A5^+^ may protect neurons from oxidative stress via the ERK1/2-mediated pathway.

### 2.5. A5^+^ Promotes N1E115 Differentiation into Dopaminergic Neurons

**Figure 7:**
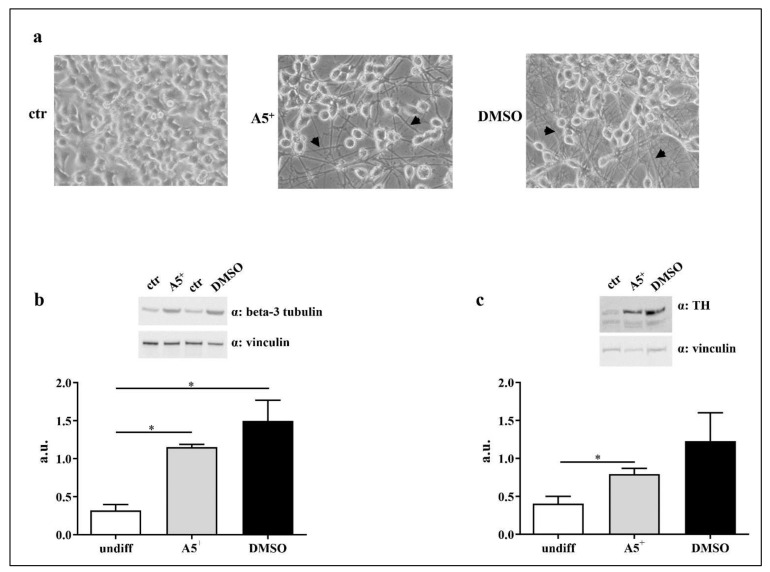
A5^+^ promotes dopaminergic neuronal differentiation. (**a**) Microscopy analysis at 20X of magnification. Black arrows indicate neurites; (**b**) Beta-3 tubulin expression. (**c**) TH steady state levels. Vinculin was used as loading control. Data are reported as mean ± SEM. Graphs represent one of three separate studies, all yielding similar results (*n* = 4). (* *p* < 0.05). ctr: control; DMSO: dimethyl sulfoxide; TH: tyrosine hydroxylase; undiff: undifferentiated; a.u.: arbitrary unit.

A loss in dopaminergic neurons is the most relevant process characterizing PD [34]. Therefore, we evaluated whether A5^+^ influences neuronal differentiation into the dopaminergic phenotype. To achieve this goal, N1E115 were differentiated as described in the Materials and Methods section. As shown in Figure 7a, A5^+^ administration induced neuronal differentiation characterized by neurites, indicated by the arrows, similarly to those promoted by DMSO. To further confirm neuronal differentiation, we analyzed the expression levels of beta-3 tubulin, a well-established marker of neuronal identity [35]. Beta-3 tubulin levels increased in both A5^+^ (*p* < 0.05) and DMSO (*p* < 0.05) treated cells, compared to control undifferentiated cells (Figure 7b), confirming that A5^+^ may promote cellular differentiation into neurons. We also tested the expression levels of tyrosine hydroxylase (TH), a well-established dopaminergic neuronal marker [36]. According to our hypothesis, TH levels were significantly enhanced following A5^+^ administration compared to control cells (*p* < 0.05), while DMSO-treated cells showed a non-significant trend. These findings suggest that A5^+^ may induce differentiation into dopamine-secreting neurons and could be helpful in restoring normal neural circuit function in PD.

## 3. Discussion

Neurodegenerative diseases are a pandemic problem worldwide [37]. All countries are employing great efforts to counteract the dramatic escalation of this phenomenon. Science already gave a lot of answers and helps in this regard; however, it is still not enough. Novel approaches, even pharmacological, natural and/or including healthy behaviors, are needed to decrease, or better, to reverse the progression of diseases, like Parkinson’s disease. In the present study, we demonstrated, innovatively, the efficacy of a polyphenols’ mixture, known as A5^+^, against an in vitro model of Parkinson’s disease. This protection was evident by the ability of A5^+^ in reducing neuroinflammation through lowering the levels of pro-inflammatory cytokines, reducing apoptosis through the inhibition of cellular death receptors, decreasing oxidative stress by the activation of ERK1/2 pathway, and promoting cellular differentiation into dopaminergic neurons. The present data suggest that, by modulating different processes implicated in Parkinson’s Diseases pathology, A5^+^ may be a useful therapeutic strategy.

We recently demonstrated that A5^+^ protected against influenza A virus and SARS-CoV-2 [22]. Specifically, A5^+^ administrated in Vero E6 cells after infection significantly blunted the influenza A virus replication and SARS-CoV-2. In SARS-CoV-2, A5^+^ was added over time and/or post-infection reduced viral replication by about two Logs, showing a strong anti-viral effect. Moreover, we reported that treatment with A5^+^ caused a reduction in IL-6 levels after viral infection [22]. These data align perfectly with present results, since here we reported a significant reduction in IL-6 after A5^+^ treatment, along with a decrease in CXCL1. Other pro-inflammatory cytokines were also diminished, including IFN-γ, IL-1β, and TNF-α. It is worth noting that a previous study in an animal model of PD, already reported, that polydatin, by passing through the blood–brain barrier, protects against motor degeneration in the *substantia nigra*, reducing the neuroinflammation induced by over-activation of microglia [38]. Similar beneficial findings have been also reported for pterostilbene when administered in a model of SH-SY5Y [39]. Moreover, by using the SH-SY5Y cell line, in a model of PD induced by rotenone, a study reported that polydatin restored neuroprotection by modulating autophagy, mitochondrial fusion and oxidative stress [16]. Intriguingly, ellagic acid has been shown to protect the brain against ROS-induced neural damage in a PD-rat model induced by 6-OHDA injected directly into the right medial forebrain bundle in lesioned rat brains [40]. In the present study, by using neuroblastoma cells, we demonstrated a dramatic reduction in apoptosis after treatment with A5^+^ by the inhibition of both intrinsic and extrinsic pathways, and by reducing the cellular death receptors. This increase in cell viability may also be linked to a direct effect of the A5^+^ on ROS production and pERK1/2 activation. The effect of zinc in neuron wellness through the ERK1/2 pathway is established [41]. We may speculate that the significant effect of A5^+^ against damage induced by 6-OHDA is due to the synergic and integrative effect of its components that act in different phases of cellular savage mechanisms. Nevertheless, A5^+^ did also induce cellular differentiation into dopamine-secreting neurons. This evidence was already suggested by the use of only polydatin in three different rodent models of PD, reporting a significant attenuation of motor defects linked to degeneration in the dopaminergic neurons in the *substantia nigra* [42]. Resveratrol has already been shown to induce protection against PD by inducing cellular differentiation into dopamine-secreting neurons [10]. Here, we innovatively investigated the neurons differentiation to test the potential translation of the compound as a therapeutic agent.

We must acknowledge limitations for the present study. The main one is that this is an in vitro study with all limitations of these kinds of analysis, which mainly include the lack of physiopathology processes. Further studies in rodents and then clinical investigation are imperative to demonstrate the utility of A5^+^ against PD. However, it is proper to report that the beneficial effects of different singular polyphenols in PD symptoms, such as spatial learning and memory skill, motor balance and coordination, and dopamine release, have been already largely reported either in in vivo models or in epidemiological studies [43].

## 4. Materials and Methods

### 4.1. Cell Culture and Treatments

Murine neuroblastoma cell lines, N1E-115, were purchased from the American Type Culture Collection (ATCC, Manassas, VA, USA) and culture in Dulbecco’s Modified Eagle Medium (DMEM) (Gibco, Thermo Fisher Scientific, Waltham, MA, USA), supplemented with 10% heat-inactivated Fetal Bovine Serum (FBS) (Corning, NY, USA) and 100 U/mL penicillin/streptomycin (Thermo Scientific, Waltham, MA, USA). Cells were maintained at 37 °C in humidified air containing 5% CO_2_.

A5^+^ was provided by SirtLife srl, Rome, Italy. A5^+^ 10 µg is composed of ellagic acid (20%), polydatin (98%), pterostilbene (20%), honokiol (20%), mixed with recommended doses of zinc, selenium, and chromium. The polydatin and A5^+^ were dissolved in DMSO (dimethyl sulfoxide, Sigma Aldrich, Milan, Italy) at 1 mg/mL [22]. In order to establish the concentration of A5^+^ able to promote beneficial effect without exerting neurotoxic effect, N1E115 cells were treated with different concentrations (10, 50, 100 and 500 μM) for 48 h.

Neurotoxicity was induced by treating cells with different concentration (10, 30, 50, 75 and 100 μM) of 6-hydroxydopamine (6-OHDA) (Sigma Aldrich, Saint Louis, MO, USA) for 24 h.

Moreover, a combined treatment has been given: cells were pre-treated with A5^+^ for 48 h and then 6-OHDA was added in combination with A5^+^ for further 24 h.

### 4.2. Cell Viability Assay and IC50 Assessment

Cell viability was determined by using the MTT (3-(4,5-dimethylthiazol-2-yl)-2,5-diphenyltetrazolium bromide) assay (Sigma Aldrich, Saint Louis, MO, USA), following manufacturer’s protocol. Briefly, 8 × 10^3^ cells/well were seeded into a 96 multi-well plate for 24 h and treated with 6-OHDA and A5^+^ as reported above. Then, medium was removed and cells were incubated for 3 h at 37 °C with 5 mg/mL of MTT. After 3 h, DMSO (dimethyl sulfoxide) (Sigma Aldrich, Saint Louis, MO, USA) was added and the formazan production was assessed by measuring the absorbance at 570 nm.

Following cell viability assay, we also assessed the IC50 (50% inhibitory concentration), by using GraphPad Prism 9 (La Jolla, CA, USA), by using the MTT values on *Y*-axis and the log of concentration on the *X*-axis.

### 4.3. Cell Differentiation

Cells differentiation was performed as reported by Oh et al. [44]. Briefly, 100.000 cells/well were seeded in a 6-well plate with normal growth medium and maintained in culture for 48 h (h). Then, the medium was changed and a differentiation medium, composed by 2% FBS + 1.25% DMSO (Sigma Aldrich, Saint Louis, MO, USA) or 10 μM of A5^+^ was added. The medium was changed every 2 days for 5 days. Then, cells were collected for further experiments.

### 4.4. Inflammatory Array

Cytokines analysis was performed by using Mouse Inflammation Array C1 (RayBiotech, Inc., Norcross, GA, USA), following the manufacturer’s protocol. Briefly, cells were treated as previously described. Then, supernatants were collected and centrifuged to precipitated cells and debris, and stored at −20 °C. Membranes with spotted antibodies were blocked with the supplied blocking buffer and incubated for 30 min at room temperature. Then, blocking buffer was removed and 1 mL of sample was placed in each well and incubated overnight at +4 °C. The day after, samples were removed and membranes were washed 3 times and subsequently incubated over night at +4 °C with biotinylated antibody cocktail. The day after the antibody was removed, membranes were washed and HRP-Streptavidin solution was added over night at +4 °C. The following day, supernatant was removed, and membranes were washed and detected by chemiluminescence. The array map is reported below (Table 1).

### 4.5. Apoptosis Assessment

For apoptosis detection, cells were stained with propidium iodide (PI) as reported by Rea et al. [45]. Briefly, N1E115 was treated as previously reported. Then, they were collected and fixed with ethanol 70% for 45 min. Subsequently, they were washed and stained with PI. Apoptotic cells, present in subG1 phase of cell cycle, were analyzed by flow cytometry.

### 4.6. Western Blot Analysis

Cells were treated as previously described. Then, they were lysed according to Pacifici et al. [46]. Briefly, cells were lysed in ice-cold buffer composed by: 20 mM Tris (pH 7.6), 137 mM NaCl, 1.5% NP40, 1 mM MgCl_2_, 1 mM CaCl_2_, Glycerol 10%, 2 mM phenylmethylsulfonyl fluoride (PMSF), phosphatase inhibitor cocktails (Sigma Aldrich, Saint Louis, MO, USA), and protease inhibitor cocktail tablets (Roche Diagnostics GmbH, Mannheim, Germany), and maintained in ice for 30 min. Then, they were centrifuged at 14,000× *g* rpm for 25 min. The collected supernatants were loaded on a 4–12% pre-cast gel (Thermo Scientific, Waltham, MA, USA) and transferred on a nitrocellulose membrane by using the Trans Turbo Blot system (Bio-Rad Laboratories, MI, Italy). Membranes were probed with the following primary antibodies: caspase-3, PARP-1, Beta-3 tubulin, Tyrosine hydroxylase, pERK1/2, total ERK1/2 and actin (1:1000 Cell Signaling Technologies, Denvers, MA, USA), vinculin (1:200, Santa Cruz Biotechnology, Santa Cruz, CA, USA).

The antigen antibody complexes were detected with enhanced chemiluminescence (ECL) reagent (GE Healthcare, Little Chalfont, UK) and quantified by using the Gel DocTM XR + with Image LabTM Software (Bio-Rad Laboratories, MI, Italy).

### 4.7. Apoptosis Array

Apoptotis analysis was performed by using Mouse Apoptosis Array C1 (RayBiotech, Inc., Norcross, GA, USA), following the manufacturer’s protocol. Briefly, cell were treated as previously described, collected and lysed by using the given Cell Lysis Buffer with Protease inhibitor. Cells were maintained on ice for 30 min and then centrifuged at 14,000× *g* rpm for 25 min at +4 °C. Supernatants were collected, the protein content was assessed by using Bradford assay (Bio-Rad Laboratories, MI, Italy), and samples were maintained in ice. Membranes with spotted antibodies were blocked with the supplied blocking buffer and incubated for 30 min at room temperature. Then, the blocking buffer was removed and 200 μg of total protein was added and incubated overnight at +4 °C. The day after, samples were removed and membranes were washed 3 times and subsequently incubated over night at 4 °C with Biotinylated Antibody Cocktail and incubated overnight at +4 °C. The day after the antibody was removed, membranes were washed and HRP-Streptavidin solution was added overnight at 4 °C. The following day, supernatant was removed, and membranes were washed and detected by chemiluminescence. The array map is reported below (Table 2).

### 4.8. ROS Production

To assess ROS production, cells were treated as previously reported. Then, the cells were treated with DCFDA 5μM for 30 min. Subsequently, the cells were centrifuged and analyzed by FACS analysis.

### 4.9. Statistical Analysis

All data were analyzed using GraphPad Prism 9 (La Jolla, CA, USA). Unpaired two-tailed Student’s test was used for statistical analysis and significance. All data were expressed as mean ± SEM, as indicated. Values of *p* < 0.05 were considered statistically significant.

## 5. Conclusions

Parkinson’s disease (PD) is the second-most common neurodegenerative disorder worldwide, and there are not yet any therapeutics or pharmacological therapies to prevent and cure this disease. It is well established that single polyphenols administration showed protective efficacy against various PD symptoms. In the present study, we demonstrated that a novel mixture of polyphenol and micronutrients known as A5^+^, by acting in a synergic manner, can counteract the noxious processes linked to Parkinson’s disease by reducing pro-inflammatory cytokine release, blunting apoptosis mechanisms, decreasing oxidative stress, and by triggering differentiation in dopamine-secreting neuronal cells. These findings suggest that A5^+^ may be a novel therapeutic strategy against Parkinson’s disease and its complications.

## Figures and Tables

**Table 1:** Inflammatory array map.

POS	POS	NEG	NEG	Blank	BLC	CD30L	Eotaxin	Eotaxin-2	Fas L	Fractalkine	GCSF
POS	POS	NEG	NEG	Blank	BLC	CD30L	Eotaxin	Eotaxin-2	Fas L	Fractalkine	GCSF
GM-CSF	IFNγ	IL-1α	IL-1β	IL-2	IL-3	IL-4	IL-6	IL-9	IL-10	IL-12	IL-12
										p40p70	p70
GM-CSF	IFNγ	IL-1α	IL-1β	IL-2	IL-3	IL-4	IL-6	IL-9	IL-10	IL-12	IL-12
										p40p70	p70
IL-13	IL-17	I-TAC	KC	Leptin	LIX	Lymphotactin	MCP-1	MCSF	MIG	MIP-1α	MIP-1γ
IL-13	IL-17	I-TAC	KC	Leptin	LIX	Lymphotactin	MCP-1	MCSF	MIG	MIP-1α	MIP-1γ
RANTES	SDF-1	TCA-3	TECK	TIMP-1	TIMP-2	TNF-α	sTNF RI	sTNF RII	Blank	Blank	POS
RANTES	SDF-1	TCA-3	TECK	TIMP-1	TIMP-2	TNF-α	sTNF RI	sTNF RII	Blank	Blank	POS

**Table 2:** Apoptosis array map.

POS	POS	NEG	NEG	BAD	BAX	BCL-2	BCL-W	BID	BIM	Caspase-3	Caspase-8
POS	POS	NEG	NEG	BAD	BAX	BCL-2	BCL-W	BID	BIM	Caspase-3	Caspase-8
CD40	CD40 Ligand	cIAP-2	CytoC	DR6	Fas	Fas-Ligand	Hsp27	Hsp60	Hsp70	HTRA2	IGFBP-1
CD40	CD40 Ligand	cIAP-2	CytoC	DR6	Fas	Fas-Ligand	Hsp27	Hsp60	Hsp70	HTRA2	IGFBP-1
IGFBP-2	IGFBP-3	IGFBP-4	IGFBP-5	IGFBP-6	IGF-1	IGF-2	p21	p27	p53	SMAC	Survivin
IGFBP-2	IGFBP-3	IGFBP-4	IGFBP-5	IGFBP-6	IGF-1	IGF-2	p21	p27	p53	SMAC	Survivin
TNF RI	TNF RII	TNF-α	TNF-β	TRAIL R2	XIAP	Blank	Blank	Blank	Blank	NEG	POS
TNF RI	TNF RII	TNF-α	TNF-β	TRAIL R2	XIAP	Blank	Blank	Blank	Blank	NEG	POS

## Data Availability

The data are contained within the article.
